# Jie-Du-Hua-Yu Granules Promote Liver Regeneration in Rat Models of Acute Liver Failure: miRNA-mRNA Expression Analysis

**DOI:** 10.1155/2020/8180959

**Published:** 2020-12-30

**Authors:** Tingshuai Wang, Na Wang, Rongzhen Zhang, Shaodong Huang, Hua Qiu, Fuli Long, Minggang Wang, Dewen Mao

**Affiliations:** ^1^School of Chinese Medicine, Hunan University of Traditional Chinese Medicine, Changsha, Hunan 410208, China; ^2^Department of Hepatology, The First Affiliated Hospital of Guangxi University of Chinese Medicine, Nanning, Guangxi 530023, China; ^3^Department of Scientific Research, The First Affiliated Hospital of Guangxi University of Chinese Medicine, Nanning, Guangxi 530023, China

## Abstract

**Purpose:**

Jie-Du-Hua-Yu (JDHY) granules are a traditional Chinese medicine with known therapeutic effects for the treatment of acute liver failure (ALF). This study explored the potential molecular mechanism(s) of JDHY granules in promoting liver regeneration and preventing ALF.

**Methods:**

Rat models of ALF were constructed through administration of D-galactosamine (D-GalN) (600 mg/kg) and lipopolysaccharides (LPS) (20 *μ*g/kg). Rats were gavaged with JDHY granules, and serum and liver samples were collected at 12 h post-D-GalN/LPS administration. The degree of liver injury was evaluated through hepatic pathology and alanine/aspartate aminotransferase (ALT/AST) activity. miRNA chips were used to detect the miRNA expression profiles of rat models. Bioinformatics analysis was used to identify the biological processes and cell signaling pathways mediating the therapeutic effects of JDHY. Real-time PCR (RT-PCR) and western blotting were used to validate the data.

**Results:**

JDHY granules could effectively decrease the levels of ALT and AST, relieve D-GalN/LPS-induced liver injury, and improve hepatic function. JDHY granules were found to regulate the expression of 20 miRNAs and 19 mRNAs, which influenced 21 biological processes and 9 signaling pathways. Upon analysis of the therapeutic mechanism(s) governing the effects of JDHY granules on liver regeneration, enhanced DNA replication and an improved cholesterol metabolic ratio were identified. JDHY granules were also found to increase the expression of MCM3, CDK4, and TC, confirming the involvement of these pathways. Moreover, JDHY granules were found to promote hepatocyte mitosis and inhibit the progression of ALF.

**Conclusion:**

JDHY granules protect against D-GalN/LPS-induced ALF in rats by promoting liver regeneration through enhanced DNA replication and an improved cholesterol metabolic ratio.

## 1. Introduction

Acute liver failure (ALF) is a life-threatening illness that is accompanied by rapid progression and poor prognosis [1]. Compared with other countries, hepatitis B virus (HBV)-related ALF has a high incidence in China, with mortality rates as high as 60 to 80% without liver transplantation [2]. The pathogenesis of ALF involves a robust immune response, hepatic necroinflammation, and decompensation [3]. However, many of the molecular mechanisms governing the occurrence of ALF remain poorly characterized.

Hepatic failure is characterized by the loss of hepatocytes [4], the regeneration of which can compensate liver function, leading to a favorable prognosis during liver failure. However, methods to promote hepatocyte regeneration in clinical medicine are currently lacking. MicroRNAs (miRNAs) are a class of noncoding RNAs of ∼19–25 nucleotides in length that bind specific target mRNAs to regulate gene expression. miRNAs are key to the control of liver function and hepatocyte proliferation [5, 6]. It has been shown that a number of miRNAs participate in cell proliferation and liver regeneration during ALF [7]. MiR-423-5p promotes cell proliferation through regulating the expression of MYC, CCNA2, and CCND1 in rat BRL-3A cells [8]. These findings highlight miRNAs as potential biological markers as well as therapeutic targets for the diagnosis and treatment of ALF.

Jie-Du Hua-Yu (JDHY) granules are a traditional Chinese medicine that consists of six Chinese herbs that has been widely used in clinical practice for decades. In clinical studies, JDHY granules show liver protective functions and can decrease the levels of serum alanine aminotransferase (ALT) and aspartate aminotransferase (AST) [9]. In vivo studies have shown that JDHY can decrease acute liver damage and downregulate the expression of caspase-3, thereby preventing hepatocyte apoptosis in rat models of ALF [10]. In this study, we investigated whether JDHY granules regulate the expression of miRNAs to exert its therapeutic benefits. D-GalN and LPS were used to establish rat models of ALF. miRNA chips were used to detect the miRNA expression profile following JDHY treatment. Bioinformatics analysis was used to identify the biological processes and pathways regulating miRNA expression. Western blot (WB) and real-time PCR (RT-PCR) were used to verify whether the genes of the related pathways were associated with liver regeneration.

## 2. Materials and Methods

### 2.1. Rat Models of ALF and Treatment with JDHY Granules

Male Wistar rats weighing 200 ± 20 g were purchased from the animal experimental center of Guangxi Medical University (Nanning, China) and housed in a pathogen-free environment. Ethical approval for the study was given by The First Affiliated Hospital of Guangxi University of Traditional Chinese Medicine Animal Experimental Ethics Committee. All rats were fed a specific pathogen-free (SPF) diet and were maintained in a room at 24 ± 3°C, with a light and dark cycle of 12 : 12 h.

D-galactosamine (D-GalN) and lipopolysaccharides (LPS) were purchased from Tianjin Chemical Co. Ltd. (Sigma, United States). ALF models were induced by a single intraperitoneal injection of D-GalN (600 mg/kg) and LPS (20 *μ*g/kg) [11]. JDHY granules manufactured by Jiangyin Tian Jiang Pharmaceutical Co. Ltd. (JiangSu, China) were purchased from the First Affiliated Hospital of Guangxi University of Traditional Chinese Medicine. The JDHY granules comprised *Artemisia capillaris* 4 g, Radix paeoniae rubrathe 7.5 g, *Rheum officinale* 5 g, *Oldenlandia diffusa* 2 g, Radix curcumae 1.5 g, and *Acorus gramineus* 3 g. Quality controls were performed as previously described [12]. Prior to use, 4 g of the dry herbal mixture was resuspended and dissolved in 1 mL of distilled water.

Rats (*n* = 30) were randomly divided into two groups: (1) model group (Model, *n* = 15)—injected with D-GalN and LPS intraperitoneally—and (2) JDHY granule group (JDHY group, *n* = 15)—injected with D-GalN and LPS intraperitoneally and gavaged with JDHY 8.8 g/kg/d. The blank control group (control, *n* = 10) was gavaged with distilled water. JDHY granules were orally gavaged for 3 days prior to modeling. Twelve hours after D-GalN and LPS administration, rats were anesthetized by the intraperitoneal injection of 3% pentobarbital sodium (45 mg/kg). Blood serum and liver samples were collected for analysis. Rats were sacrificed by cervical dislocation.

### 2.2. Ethical Approval and Consent to Participate

Ethical approval for the study was provided by The First Affiliated Hospital of Guangxi, University of Traditional Chinese Medicine.

### 2.3. Serum Biomarkers for Liver Failure

Collected blood samples were coagulated for 2 h, and the upper pale yellow serum layer was collected and centrifuged at 12000 g at 4°C for 15 min. Supernatants were collected, and serum ALT and AST activities were assessed via ELISA assays (Wuhan Huamei Bioengineering Institute, Wuhan, China).

### 2.4. Liver Histopathological Examinations

Sections of rat livers were fixed in 4% paraformaldehyde for 24 h and embedded in paraffin. Wax blocks of liver tissue were sliced into sections of ∼3 *μ*m thickness and stained with hematoxylin and eosin (H&E). Sections were imaged under a light microscope (Olympus, Japan).

### 2.5. Detection of Liver Regeneration

JDHY-treated liver tissues were minced using a scalpel in PBS, filtered through a wire mesh screen (#100 mesh), and stained with propidium iodide to assess their cell cycle status by flow cytometry (FCM). Cell cycle phases were analyzed on a Becton–Dickinson FCM Calibur. PI was calculated by the formula: PI = (S + G2M)/(G0/1 + S + G2M) × 100%. The mitotic index (MI) was measured by hematoxylin-eosin (HE) staining. The number of mitotic cells in a sample of ∼2000 liver cells was quantitated by microscopy. Nuclear division was simultaneously determined by two pathologists. MI = mitotic cell count/2000 × 100% [13].

### 2.6. miRNA and mRNA Expression Arrays

Total RNA was isolated from the liver samples using miRNeasy Mini Kits (QIAGEN, 217184). RNA integrity was determined using an Agilent 2100 Bioanalyzer (Agilent Technologies, CA, USA). Affymetrix GeneChip miRNA 4.0 Arrays (Affymetrix, CA, USA) were used to determine miRNA expression profiles. Total RNA was used to detect genome-wide expression. Affymetrix GeneChip 1.0 Arrays were used to determine the mRNA expression profile. Affymetrix CEL files were imported into GCBI (https://www.gcbi.com.cn) for screening and assessment of expression profiles.

### 2.7. miRNA Target, mRNA Prediction, and Functional Enrichment

TargetScan (http://www.targetscan.org) and miRanda (http://www.microrna.org/microrna/home.do) were used to identify mRNA targets of the miRNAs [14]. ToppCluster (http://toppcluster.cchmc.org) was used for functional enrichment analysis [15]. Cytoscape software was used to design the miRNA-mRNA regulatory and functional enrichment network [16].

### 2.8. RT-PCR Analysis

Quantitative real-time PCR was used for the detection of miRNA and RNA expressions. Trizol Reagent (Invitrogen, China) was used to isolate total RNA from the liver samples of each group. SuperScript III Reverse Transcriptase (Invitrogen, China) was used to synthesize cDNA, which was amplified using SYBR Green qPCR Mix (Invitrogen, China). The ABI 7,500 qPCR system was used to detect potential target miRNAs and mRNAs. U6 and *β*-actin were used as internal controls for miRNAs and mRNAs, respectively. Relative gene expression was assessed using the 2^–△△^CT method [17].

### 2.9. Western Blot Analysis

Liver tissues were harvested in lysis buffer (Solarbio, Beijing, China) and resolved through SDS-PAGE electrophoresis. Total protein was then transferred onto 0.22 **μ**m PVDF membranes and probed with primary antibodies against MCM3 (1 : 6000, ERP7080; Abcam), *β*-actin (1 : 6000, ab8227; Abcam), GAPDH (1 : 6000, ab181602; Abcam), CDK4 (1 : 6000, ab137675; Abcam), p38 MAPK (1 : 6000, ab31828; Abcam) and the appropriate species-specific HRP-conjugated secondary antibodies. Chemiluminescence detection was performed using the ECL Western Blotting Substrate (PEoo10; Solarbio). The Quantity One system (Bio-Rad) was used for analysis.

### 2.10. Statistical Analysis

miRNA and mRNA data were analyzed using the Robust Multichip Average (RMA) algorithm as a normalization method. Fold changes were used to identify differentially expressed genes (DEGs). Specific DEGs were selected according to the fold-change threshold. The association strength of miRNAs and gene functions was determined using a Fisher's exact test. Real-time PCR data were analyzed using *t*-tests. Statistical significance was set at *P* < 0.05.

## 3. Results

### 3.1. Serum Biochemistry and Liver Histopathology in Rats

**Figure 1:**
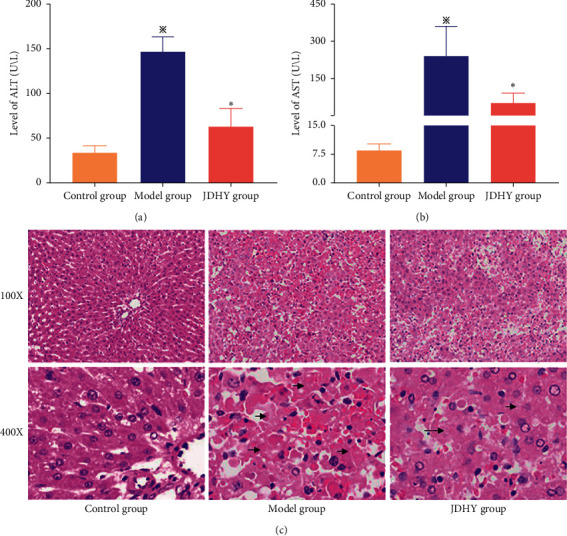
Serum biochemistry and liver histopathology in rat ALF models. (a) Serum ALT. (b) Serum AST. Hematoxylin and eosin staining of liver sections (100x and 400x) of (c) control, (d) model, and (e) JDHY groups. ^※^*P* < 0.05 vs. control group, ^*∗*^*P* < 0.05 vs. model group. Islet structures are marked with arrows (⟶). *n* = 10 in the control group, *n* = 7 in the model group, and *n* = 10 in the JDHY group. Abbreviations: ALT, alanine aminotransferase; AST, aspartate aminotransferase; JDHY, Jie-Du-Hua-Yu.

After D-GalN and LPS administration, 7 of 15 rats survived in the control group, compared with 10 of 15 rats in the JDHY group. As shown in Figure 1(a), the serum levels of ALT in the model group and JDHY group were higher than the control group (*t* = 20.13, 4.35, *P* < 0.05). Compared with the model group, the ALT levels were lower in the JDHY group (*t* = 10.43, *P* < 0.05). As shown in Figure 1(b), the serum levels of AST in the model group and JDHY group were higher than that in the control group (*t* = 6.614, 5.156, *P* < 0.05). Compared with the model group, the JDHY group had lower AST levels (*t* = 4.72, *P*=0.002).

HE staining showed that the morphology of liver tissue in the control group was normal, the hepatic lobule structure was complete, the hepatic cell cords were arranged radially, and the nuclear structure was clear (Figure 1(c)). Liver histological observations showed serious liver injury in the model group in which liver tissue was extensively necrotic, the tissue structure was seriously damaged, and red blood cell filling and inflammatory cell infiltration were increased (Figure 1(d)). In the JDHY group, the liver tissue structure was damaged to a moderate degree, but cells were arranged in order, with fewer red blood cells and inflammatory cells visible (Figure 1(e)).

### 3.2. JDHY Granules Promote Liver Regeneration

**Figure 2:**
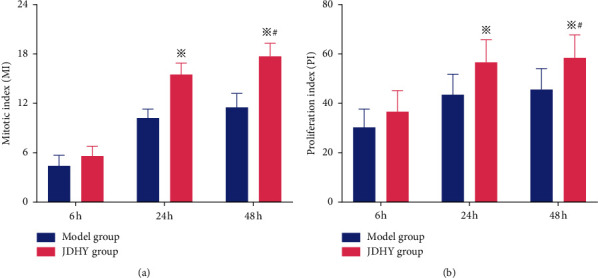
MI and PI levels after treatment with JDHY granules in rats with ALF. (a) MI and (b) PI. ^※^*P* < 0.05 vs. model group, ^#^*P* < 0.05 vs. 24 h. Abbreviations: PI, proliferation index; MI, mitosis index.

In the previous studies, the proliferation index (PI) and mitosis index (MI) of JDHY-treated rats were significantly higher than the model group at 24 and 48 h (*P* < 0.05) (Figure 2). Based on these data, JDHY granules were shown to promote the proliferation and division of hepatocytes in rats with ALF. The mechanism of action of JDHY granules reported was related to enhanced DNA replication in the S phase [13].

### 3.3. Changes in miRNA Expression after JDHY Treatment in Rat ALF Models

**Figure 3:**
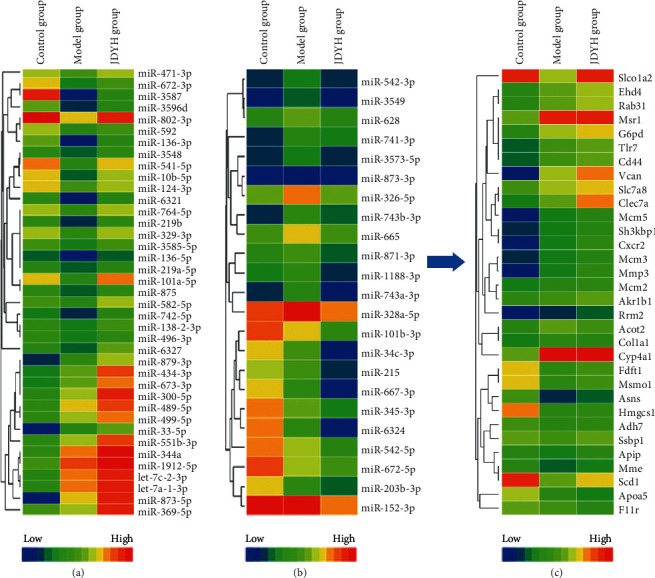
Selected miRNAs and potential target genes after treatment with JDHY granules. (a) 23 downregulated miRNAs, fold change ≤ −2 (*P* < 0.05). (b) 39 upregulated miRNAs, fold change ≥2 (*P* < 0.05). HemL software was used to depict the expressional changes of the miRNAs after treatment with JDHY granules. Columns represent the expression levels of each miRNA. Changes from low to high are depicted as a color variation from blue to red. (c) TargetScan and miRanda were used to predict miRNA target genes (the number of downregulated miRNAs: 23; the number of upregulated miRNAs: 39). Data were combined with the mRNA profile to select genes that changed ≥1.5 fold or ≤1.5 fold. According to the miRNA-mRNA expression pattern to select target genes (miRNA downregulated and mRNA upregulated, miRNA upregulated and mRNA downregulated), 32 genes were selected as potential target genes. HemL software was used to depict the expressional changes of each mRNA. Each column represents the mRNA expression level. Fold changes from low to high are depicted as color variations from blue to red.

To identify the miRNAs regulated by JDHY granules in rat models of ALF, the miRNA expressional profiles were compared between control, model, and JDHY groups using miRNA chips. We selected miRNAs according to fold changes ≥2 or ≤−2 fold (*P* < 0.05) in both the model and JDHY groups. In total, 23 miRNAs were downregulated and 39 miRNAs were upregulated by the JDHY granules. Heatmaps were used to show changes in the miRNA expression profiles of each group (Figures 3(a) and 3(b)).

### 3.4. Expression of Target Genes in the JDHY Group

Genome-wide expression profiles were assessed using mRNA chips in the control group, model group, and JDHY group. A list of 62 miRNA target genes (downregulated = 23; upregulated = 39) were compiled based on TargetScan and miRanda predictions. We combined with the gene expression platform to select target genes with fold changes ≥1.5 or ≤1.5 fold, *P* < 0.05. Moreover, according to the miRNA-mRNA expression pattern of select target genes (miRNA downregulated mRNA upregulated, miRNA upregulated mRNA downregulated), 32 genes were identified as potential targets. Heatmaps were used to show the changes in expression levels for these genes (Figure 3(c)).

### 3.5. Target Genes and Functional Enrichment

**Figure 4:**
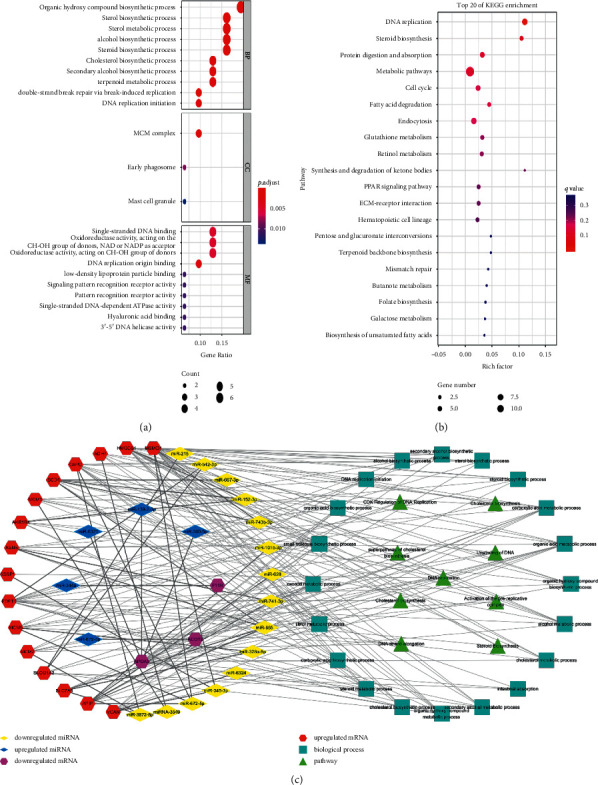
GO and KEGG enrichment analysis of the potential targets of JDHY granules in ALF. (a) GO enrichment analysis of the potential targets, BP (biological processes), MF (molecular functions), and CC (cellular components). (b) KEGG enrichment analysis of the potential targets (top 20). (c) Network of miRNA and mRNA coexpression and mRNA function enrichment. Abbreviations: GO, Gene Ontology; KEGG, Kyoto Encyclopedia of Genes and Genomes.

Gene Ontology (GO) and Kyoto Encyclopedia of Genes and Genomes (KEGG) enrichment analysis were performed using R language to detect the biological functions of the 32 potential targets, in addition to their associated pathways (Figures 4(a) and 4(b)). The ToppCluster platform was used to enrich for biological processes and pathways. Cytoscape software was used to draw the miRNA-mRNA coexpression network. Our data showed that the 19 target genes were significantly enriched in 21 biological processes and 9 pathways. Moreover, 20 miRNAs were coexpressed with the 19 target genes (Figure 4(c)). These included 5 miRNAs that were upregulated and 3 target genes that were downregulated, and 15 miRNAs that were downregulated and 16 target genes that were upregulated.

### 3.6. JDHY Granules Enhance DNA Replication

**Figure 5:**
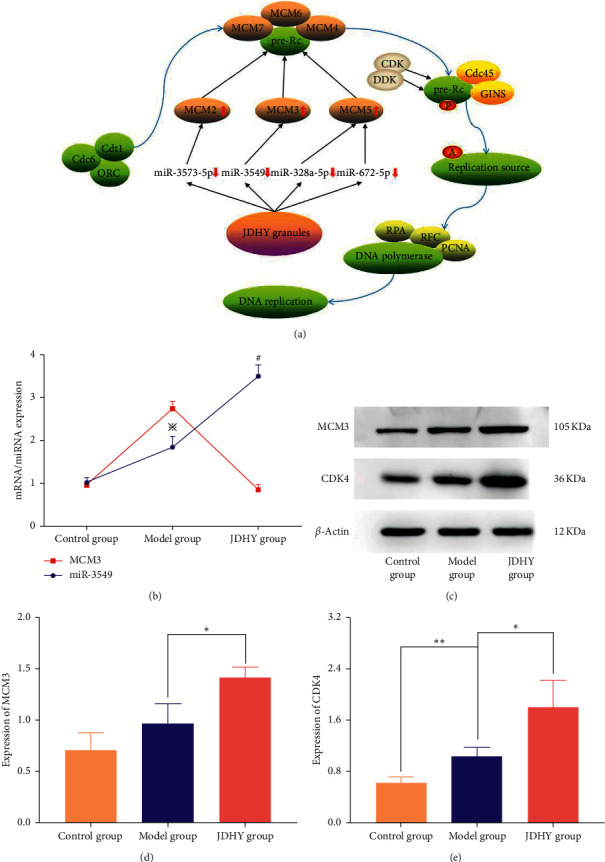
JDHY granules enhance DNA replication. (a) Therapeutic mechanisms of the JDHY granules in enhancing DNA replication. (b) mRNA/miRNA expression of MCM3/miR-3549. (c) Western blot analysis of MCM3 and CDK4. *β*-actin was probed as loading control. (d) Expression of MCM3 and (e) CDK4. *n* = 10 in the control group; *n* = 7 in the model group; *n* = 10 in the JDHY group. Abbreviations: P, phosphorylation; A, activation; ↓, downregulation; ↑, upregulation; MCM3, minichromosome maintenance complex3; CDK4, cyclin-dependent kinase 4.

Following KEGG pathway analysis, we examined the relationship between the miRNAs and mRNAs in terms of DNA replication. The JDHY granules downregulated the expression of miR-3573-5p, miR-3549, miR-328a-5p, and miR-672-5p and upregulated the expression of MCM2, MCM3, and MCM5. In addition, the JDHY granules enhanced the synthesis of pre-replication complex (pre-RC) (Figure 5(a)). The expression of MCM3 was regulated by the JDHY granules through miR-3549 (Figure 5(b)). Compared with the model group, the expression of MCM3 and CDK4 increased in the JDHY group (*P* < 0.05) (Figures 5(c)–5(e)).

### 3.7. JDHY Granules Improve the Cholesterol Metabolic Ratio

**Figure 6:**
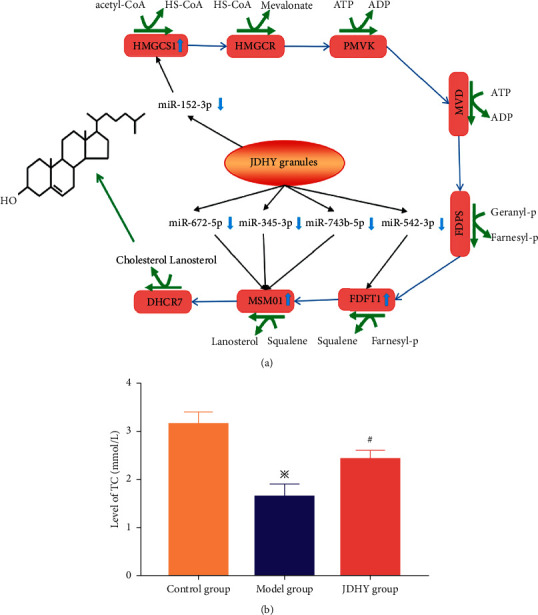
JDHY granules improve the cholesterol metabolic ratio. (a) Therapeutic mechanisms of JDHY granules in improving the cholesterol metabolic ratio. (b) Levels of TC each group. *n* = 10 in the control group; *n* = 7 in the model group; *n* = 10 in the JDHY group. Abbreviations: TC, total cholesterol.

The JDHY granules downregulated miR-542-3p, miR-152-3p, miR-345-3p, miR-672-5p, and miR-743b-3p expressions; upregulated FDFT1, HMGCS1, and MSMO1; and promoted the conversion of acetyl-CoA to HS-CoA (coenzyme-a in tissue oxidation), farnesyl-P (farnesyl pyrophosphate) to squalene, and squalene to lanosterol (2,3-oxidosqualene-lanosterol cyclase) (Figure 6(a)). Compared with the model group, the levels of total cholesterol (TC) were higher in the JDHY group (*P* < 0.05) (Figure 6(b)).

## 4. Discussion

Following the analysis of the expression profiles of miRNAs and mRNAs in the liver tissues of ALF rats, we identified 23 downregulated miRNAs and 39 upregulated miRNAs following JDHY granule treatment (Tables 1 and 2), revealing them as novel therapeutic targets. By constructing the miRNA, mRNA, biological processes, and pathways network, a total of 21 biological processes were influenced by the JDHY granules, through the regulation of 20 miRNAs. These included the upregulation of miR-344a, miR-6321, miR-300-5p, miR-672-3p, and miR-138-2-3p and the downregulation of miR-215, miR-667-3p, miR-542-3p, miR-152-3p, miR-101b-3p, miR-743b-3p, miR-741-3p, miR-628, miR-3549, miR-6324, miR-665, miR-672-5p, miR-3573-5p, miR-328a-5p, and miR-345-3p. In addition, the mRNA expression levels of 19 target genes were specifically related to 9 pathways. Upon further analysis, these pathways could be divided into DNA replication (including activation of the pre-replicative complex) and cholesterol biosynthesis (including steroid biosynthesis).

DNA replication is essential to cell regeneration [18]. During DNA synthesis, the origin recognition complex (ORG) must interact with DNA to form a pre-replication complex (pre-RC) [19]. Cdc6, Cdt1, and minichromosome maintenance proteins 2–7 (MCM2-7) then complete the assembly of the pre-RC [20]. Cyclin-dependent kinase (CDK) and Dbf4-dependent kinase (DDK) are then activated by the pre-RC through the phosphorylation of a series of PRC proteins. The replication source is then activated by the assembly of Cdc45-MCM-GINS complex (CMG complex) following cell division, and cycle 45 (Cdc45) and Go-Ichi-Ni-San (GINS) are recruited to the pre-RC [21]. Finally, replication protein A (RPA), replication factor C (RFC), proliferating cellular nuclear antigen (PCNA), and DNA polymerase bind to the activated pre-RC to initiate the process of DNA replication [22].

DNA replication is a vital biological event during the S phase of mitosis [23]. In rat models of ALF, DNA replication is essential for hepatic regeneration [18]. MCM3, a key component of the heterohexameric minichromosome maintenance (MCM) complex, plays an important role in mammalian DNA helicase activity and the initiation of DNA replication [24]. Similarly, CDK4 plays a key role in cell proliferation, particularly during the progression of cells from the G1 to S phase of the cell cycle [25]. It has been previously reported that the p38 MAPK pathway, TNF-alpha pathway, and IL-6 pathways are involved in liver regeneration through their regulation of DNA replication [26, 27]. DNA replication is also regulated by miRNAs, including the miRNA-34 family and miRNA-1269 through targeting MCM2-7 mRNA [28–30]. In this study, we demonstrated that JDHY granules can affect DNA replication by upregulating MCM2, MCM3, and MCM5 via the downregulation of miR-3573-5p, miR-3549, miR-328a-5p, and miR-672-5p (Figure 5(a)). Moreover, the expression of the cell proliferation-associated proteins CDK4 and MCM3 increased following treatment with the JDHY granules (Figures 5(b)–5(e)). These findings are the first to identify miRNAs as the therapeutic targets through which JDHY granules enhance DNA replication. In addition, the levels of MCM3 and CDK4 in the model group increased within 12 h of the onset of ALF. This is mainly because the liver itself has a strong capacity to regenerate. Even during ALF, the repair mechanisms of liver tissue are activated, even during hepatocyte death [31, 32]. Therefore, the expression of MCM3 and CDK4 in the model group increased.

As one of the major components of cell membranes and as a precursor for an array of biologically active molecules, cholesterol plays an important physiological roles in vivo [33]. Cholesterol synthesis must be regulated to meet the physiological needs of the cell. Cholesterol synthesis can be divided into be five major stages: (1) conversion of acetyl-CoA to 3-hydroxy-3-methylglutaryl-CoA (HMG-CoA); (2) conversion of HMG-CoA to mevalonate; (3) conversion of mevalonate to the isoprene-based molecule isopentenyl pyrophosphate (IPP), with the concomitant loss of CO2; (4) conversion of IPP to squalene; and (5) conversion of squalene to cholesterol [34].

Cholesterol homeostasis is key to liver function. Liver-specific HMGCR knockout mice display hepatic-associated death with jaundice and hypoglycemia [35]. Low levels of cholesterol have been detected in rat models of ALF, which correlates with disease severity in ALF patients [36, 37]. Bile acid is a cholanic acid in bile, which is synthesized from cholesterol in the liver. Recent studies have shown that bile acids act as signaling molecules and play important regulatory roles in liver regeneration through their activation of signaling pathways, such as the p38 MAPK signaling pathway [38]. Cholesterol metabolism can be regulated by miRNAs [39], including miRNA-122 and miRNA-33 that target the sterol response element binding protein 2 (SREBP-2) that plays a major role in the regulation of cholesterol homeostasis and metabolism. In this study, we found that JDHY granules can improve the cholesterol metabolic ratio by upregulating FDFT1, HMGCS1, and MSMO1 and by downregulating miR-542-3p, miR-152-3p, miR-345-3p, and miR-672-5p (Figure 6). The results strongly indicate that the therapeutic mechanism of JDHY granules involves improving the cholesterol metabolic ratio.

## 5. Conclusions

In summary, using integrative bioinformatics to select miRNA and mRNA expression profiles in rat models of ALF, we found that JDHY granules regulate 20 miRNA expression targets and 19 mRNAs, which influence 21 biological processes and 9 signaling pathways. Moreover, our results demonstrated that JDHY granules can protect against D-GalN/LPS-induced ALF in rats by promoting liver regeneration through enhancing DNA replication and restoring the cholesterol metabolic ratio.

## Figures and Tables

**Table 1:** List of miRNAs downregulated after JDHY treatment (fold change ≤−2, *P* < 0.05).

Transcript ID	Model vs. control	JDHY vs. model
	Fold change	Fold change
miR-542-3p	2.26	−2.26
miR-3549	2.23	−2.23
miR-741-3p	4.25	−2.10
miR-628	4.47	−4.47
miR-871-3p	2.14	−4.94
miR-3573-5p	2.65	−2.65
miR-873-3p	2.07	−2.13
miR-743b-3p	3.97	−3.04
miR-743a-3p	3.14	−4.92
miR-328a-5p	2.20	−3.67
miR-1188-3p	2.06	−5.64
miR-326-5p	3.17	−3.31
miR-665	4.68	−3.66
miR-101b-3p	−2.30	−4.88
miR-672-5p	−3.85	−2.52
miR-203b-3p	−4.26	−3.93
miR-542-5p	−2.15	−7.63
miR-215	−3.14	−6.42
miR-345-3p	−3.80	−6.23
miR-152-3p	−2.11	−2.09
miR-34c-3p	−3.52	−14.31
miR-6324	−8.11	−15.29
miR-667-3p	−4.62	−14.43

**Table 2:** List of miRNAs upregulated after JDHY treatment (fold change ≥2, *P* < 0.05).

Transcript ID	Model vs. control	JDHY vs. model
	Fold change	Fold change
miR-471-3p	−2.05	2.41
miR-672-3p	−8.67	2.57
miR-6327	−2.26	6.24
miR-3548	−3.26	2.82
miR-6321	−4.78	3.25
miR-764-5p	−2.87	2.82
miR-219b	−3.73	3.73
miR-219a-5p	−2.03	2.03
miR-10b-5p	−11.81	8.04
miR-3587	−71.98	6.75
miR-101a-5p	−5.56	6.43
miR-802-3p	−4.32	2.42
miR-3596d	−6.77	2.65
miR-582-5p	−2.38	3.74
miR-3585-5p	−2.38	2.38
miR-124-3p	−2.48	2.31
miR-136-3p	−10.16	3.75
miR-592	−4.36	2.37
miR-875	−2.57	2.72
miR-541-5p	−7.16	5.45
miR-329-3p	−2.15	2.15
miR-742-5p	−2.56	3.09
miR-138-2-3p	−2.20	2.66
miR-496-3p	−2.11	2.53
miR-136-5p	−3.25	3.25
miR-879-3p	3.83	2.51
miR-434-3p	4.47	2.99
miR-344a	6.87	2.69
miR-1912-5p	4.75	2.11
miR-673-3p	3.53	2.54
miR-489-5p	2.79	2.29
miR-873-5p	21.13	3.05
miR-369-5p	2.10	3.35
miR-551b-3p	2.63	2.62
miR-499-5p	2.43	2.03
let-7c-2-3p	6.05	2.22
miR-33-5p	5.26	2.82
miR-300-5p	4.37	2.85
let-7a-1-3p	6.05	2.22

## Data Availability

Data are available upon request to the corresponding authors.
